# Inadequate reporting quality of registered genome editing trials: an observational study

**DOI:** 10.1186/s12874-022-01574-0

**Published:** 2022-05-02

**Authors:** Diana Jurić, Michael Zlatin, Ana Marušić

**Affiliations:** 1 Department of Pharmacology, School of Medicine, University of Split, Šoltanska 2, 21000 Split, Croatia; 2 School of Medicine, University of Split, Šoltanska 2, 21000 Split, Croatia; 3 Department of Research in Biomedicine and Health, School of Medicine, University of Split, Šoltanska 2, 21000 Split, Croatia

**Keywords:** Clinical trials on genome editing as topic, Genome editing, Databases, Reporting

## Abstract

**Background:**

To assess registration completeness and safety data of trials on human genome editing (HGE) reported in primary registries and published in journals, as HGE has safety and ethical problems, including the risk of undesirable and unpredictable outcomes. Registration transparency has not been evaluated for clinical trials using these novel and revolutionary techniques in human participants.

**Methods:**

Observational study of trials involving engineered site-specific nucleases and long-term follow-up observations, identified from the WHO ICTRP HGE Registry in November 2020 and two comprehensive reviews published in the same year. Registration and adverse events (AEs) information were collected from public registries and matching publications. Published data were extracted in May 2021.

**Results:**

Among 81 eligible trials, most were recruiting (51.9%) phase 1 trials (45.7%). Five trials were withdrawn. Most trials investigated CAR T cells therapies (45.7%) and used CRISPR/Cas9 (35.8%) ex vivo (88.9%). Among 12 trials with protocols both registered and published, eligibility criteria, sample size, and secondary outcome measures were consistently reported for less than a half. Three trials posted results in ClinicalTrials.gov, and one reported serious AEs.

**Conclusions:**

Incomplete registration and published data give emphasis to the need to increase the transparency of HGE trials. Further improvements in registration requirements, including phase 1 trials, and a more controlled publication procedure, are needed to augment the implementation of this promising technology.

**Supplementary Information:**

The online version contains supplementary material available at 10.1186/s12874-022-01574-0.

## Background

Human gene therapy (GT) products are biological products that have a potential to fulfill unmet medical needs [1] but are also challenging to regulators because of highly complex information on their development and manufacture [1–4]. According to the FDA consideration, “human gene therapy products” are defined as „all products that mediate their effects by transcription or translation of transferred genetic material, or by specifically altering host (human) genetic sequences “ [3]. The development of innovative approaches, including programmable nucleases [5–7], led to the explosion of interest for use of genome editing [8], and a move beyond the basic laboratory research to early clinical uses [5, 8–13]. The term “genome editing” refers “to the processes by which the genome sequence is changed by adding, replacing, or removing DNA base pairs” [3, 8].

Despite their potential for different diseases [14, 15], there are still emerging issues surrounding the safety and effectiveness of GT, including early failures [1], outcomes such as death [16], late-onset T-cell leukemia [17], and brain and spinal cord tumors [18]. Due to possible non-specific off-target genome changes, insertional mutagenesis with integrating vectors, or immune response to product components, participants in trials with genome editing-based GT products may experience unpredictable and delayed adverse events (AEs) [8, 19]. To better interpret that risk, monitoring of long-term safety is recommended [19, 20]. Although AEs are mandated to be reported for all interventional trials except phase 1 involving FDA-regulated drug, biological, or device products [21, 22], reporting transparency is still low [23–25].

The aim of our study was to assess the registration completeness and published data in journal articles for trials testing genome editing therapies.

## Methods

### Sample and inclusion criteria

On November 12, 2020, we retrieved clinical trials using genome editing technologies from the Human Genome Editing (HGE) Registry provided by the WHO International Clinical Trials Registry Platform (ICTRP) [26], without any set limitations. We also checked two comprehensive tables published in 2020 [5, 9] on trials that involved genome editing. A clinical trial was considered a “HGE trial” if it: 1) had a registry identification number and available registration protocol, 2) was registered on or before November 12, 2020, and 3) involved in vivo or ex vivo interventions using engineered site-specific nucleases to alter human cells for purposes of treating or preventing disease, or was 4) long-term follow-up (LTFU) observation with extended assessments. Basic laboratory studies on human cells or tissues focusing on cellular, molecular, biochemical, genetic, or immunological mechanisms, and duplicate trials registered in two or more registries, were excluded from the analysis.

### Publication search

Corresponding publications were identified in May 2021 by screening the following sources: 1) the Publications subheading under the ClinicalTrials.gov Descriptive Information heading, 2) PubMed/MEDLINE, and 3) Scopus. The manual search used 1) trial unique identification number, and 2) combination of search terms for each trial: intervention name, nuclease platform used, edited gene/cells, condition, study phase, and all names under “investigators” field in different public registries.

### WHO ICTRP data extraction

We extracted data on 21 out of 24 items from the WHO Trial Registration Data Set (TRDS) [27] until February 2021. Administrative items (3, 7 and 8) were not extracted. We also extracted data describing HGE technology (edited cells, target gene, nuclease platform used, and modes of delivery).

For trials with posted results, we calculated the median time for results reporting from the primary completion date (PCD) [22]. In May 2021, we re-evaluated whether trials posted results in the registry.

### Data analysis

Registration and publication data were extracted independently by two reviewers (M.Z., D.J.), after all three investigators (M.Z., D.J., A.M.) established the final extraction protocol through the pilot extraction of a 10% random sample. The data are presented as frequencies and medians with 95% confidence intervals. MedCalc version 20.008 (MedCalc Software, Ostend, Belgium) was used.

## Results

### General characteristics of registered HGE trials

**Fig. 1:**
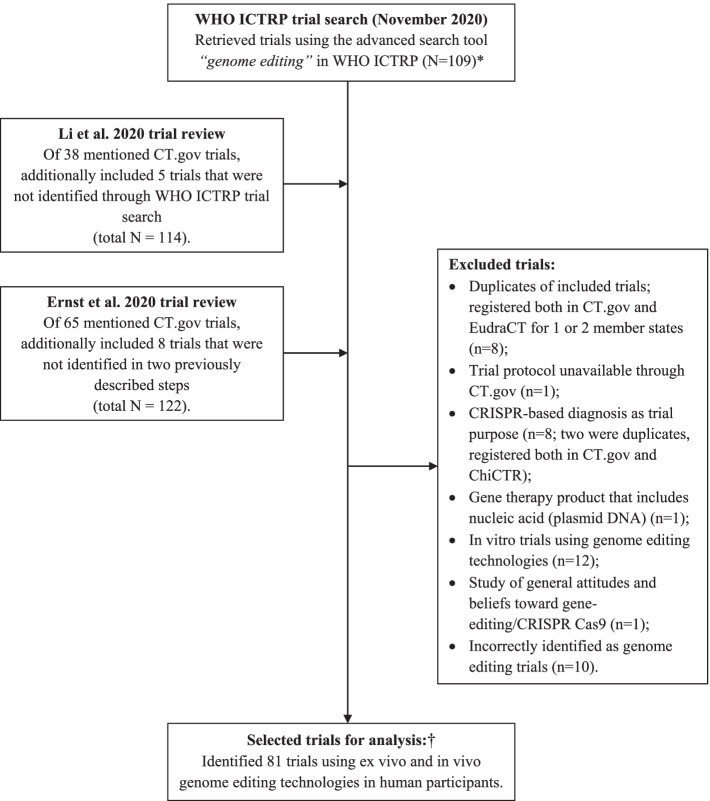
Flow-chart of the search and selection of eligible clinical trials using genome editing technologies. *For this observational study “human GT products” were defined according to the FDA as „all products that meet the definition of biological products and that mediate their effects by transcription or translation of transferred genetic material, or by specifically altering host (human) genetic sequences “ [3]. The term “human genome editing” is used “to refer to the processes by which the genome sequence is changed by adding, replacing, or removing DNA base pairs” [3, 8]. †Of the remaining 81 trials, 21 (25.9%) were identified only in WHO ICTRP, 47 (58.0%) also in published review(s), and 13 (16.0%) in only published review(s). Considering primary registries in the WHO registry network, 62 trials (76.5%) were registered only in ClinicalTrials.gov, 6 (7.4%) both in ClinicalTrials.gov and EU Clinical Trials Register (EU-CTR) (2 trials had equal protocol registered for 2 EU member states), 1 (1.2%) only in EU-CTR, and 12 (14.8%) trials only in Chinese Clinical Trial Registry (ChiCTR)

From 122 identified trials (Fig. 1), we excluded 41 (33.6%) because of in vitro trial design, incorrect identification, clustered regularly interspaced short palindromic repeats (CRISPR)-based diagnosis as trial purpose, and registration of the same trial in two registries. Of the remaining 81 trials, most were interventional (96.3%), with single group assignment as the most common model (60.3%, Table 1). The majority of trials with administered interventions were without blinding (78.2%), a half were in phase 1 (47.4%), and 25 interventional trials (32.1%) reported the use of a FDA-regulated drug product under their study intervention. The use of the randomization method was reported in 6 ClinicalTrials.gov trials that had parallel design, but detailed information regarding the randomization process, mostly challenging to these trials, were missing.

**Table 1:** Design of 81 trials on genome editing in humans registered in WHO ICTRP

Trial design features	No. (%) of trials
**Study type:**	
Interventional^a^	78 (96.3)
Long-term follow-up^b^	3 (3.7)
**Primary purpose:**^c^	
Treatment^d^	63 (80.8)
Other	2 (2.6)
Not provided^e^	13 (16.7)
**Study phase:**^c^	
Phase 0	3 (3.8)
Early phase 1	4 (5.1)
Phase 1	37 (47.4)
Phase 2	7 (9.0)
Phase 1/2	20 (25.6)
Stated “not applicable”	6 (7.7)
Not provided^f^	1 (1.3)
**Allocation:**^c^	
Randomized controlled trial^g^	6 (7.7)
Non-randomized trial	23 (29.5)
Not precisely stated, but single arm reported	7 (9.0)
Stated “not applicable”	38 (48.7)
Not provided	4 (5.1)
**Intervention study model:**^c^	
Single group	47 (60.3)
Parallel	14 (17.9)
Sequential	15 (19.2)
Not provided	2 (2.6)
**Masking:**^c^	
Open-label	61 (78.2)
Single-blind^h^	3 (3.8)
Double-blind^i^	1 (1.3)
Stated “not applicable”	5 (6.4)
Not provided	8 (10.3)
**Placebo:**^c^	
Placebo comparator noted^j^	3 (3.8)
Not provided	75 (96.2)

*Abbreviation: WHO ICTRP* World Health Organization International Clinical Trial Registry Platform

^a^Among 78 trials, 11 (14.1%) were incorrectly classified as observational in their registration protocol (1 trial from CT.gov, NCT02867345, and 10 trials from ChiCTR), and 1 (1.3%) ChiCTR trial had “Cause/Relative factors study” stated under its study type (ChiCTR1800019378)

^b^NCT04208529 was registered as an observational study to evaluate the long-term safety and efficacy in subjects who received CTX001 in 2 trials (NCT03655678 and NCT03745287) analyzed in this study as interventional trials, whilst other 2 LTFU trials, NCT04201782 and NCT02735083, had an interventional study type in their registration protocol and did not specify to which trials they referred to

^c^Characteristics related only to trials with an administered intervention (*n* = 78)

^d^One trial had “therapy” among registered terms under the trial scopes in EudraCT

^e^Among these 13 trials, 12 were registered in the Chinese trial registry, without the specific field for primary purpose, and 1 trial had an inappropriately registered observational type in CT.gov (NCT02867345)

^f^CT.gov trial mentioned previously (NCT02867345). This trial is among trials without provided data for all parameters in the table signed with the superscript “c”

^g^One parallel ClinicalTrials.gov trial reported “randomized” under the Study Design field used for data analysis, but under the Detailed Description field also reported “non-randomized” (NCT03298828)

^h^Three trials from ClinicalTrials.gov (NCT03525652, NCT03525782, and NCT03706326)

^i^One ClinicalTrials.gov trial (NCT03666871)

^j^Two trials were open-label (NCT02863913 and NCT02867332), and one was single-blind (NCT03525782)

In 3 trials that clearly noted the use of a sham control, patient’s lymphocytes were collected and infused back to the patients without any genetic or engineered modification ex vivo.

Three trials were registered as LTFU trials, referring to subjects treated with CCR5-zinc-finger nuclease (ZFN) modified autologous T cells, CRISPR/CRISPR-associated protein (Cas) 9 modified autologous hematopoietic stem cells, and UCART19, respectively.

Most trials included participants of both genders (91.4%, Supplementary Table 1), aged 18–70. Among 7 trials that investigated HGE technology in a single gender, 1 explicitly provided a “gender eligibility description” (NCT03525652).

Among 52 trials that had any registration entry for individual participant data (IPD) sharing statement, 22 trials (42.3%) will not share de-identified IPD. Regarding the data monitoring committee, 28 trials (34.6%) stated “no”, 43 (53.1%) stated “yes”, and 10 (12.3%) did not provide any information.

More than half of the trials had a “recruiting” status (51.9%) and were prospectively registered (67.9%, Supplementary Table 2). For almost a third of trials, the information on investigators were absent (30.9%). Most HGE trials were sponsored by industry (44.4%) and conducted in China (48.1%). Among 33 trials whose study completion date (SCD) was until May 2021 (median 2019, 95% CI 2018–2020, range 2013–2021), 3 trials (9.1%) had a “not yet recruiting” status, whilst 7 (21.2%) were still recruiting.

In 2017 or 2018, 4 trials changed their status to “unknown” from “not yet recruiting”, “recruiting”, or “active, not recruiting”, without any explanation. Among 5 trials that were withdrawn, 3 listed “no funding” as an explanation, 1 listed “sponsor's decision and not a consequence of any safety concern”, and 1, led by Chinese scientist He Jiankui (ChiCTR1800019378), stated that “the original applicants cannot provide the IPD for reviewing.”

Only 3 trials from ClinicalTrials.gov submitted and posted their results until May 2021 (Supplementary Table 2); results reporting times were 45.5, 31.8 and 28.0 months, respectively. All 3 trials, testing editing of CCR5 or PDCD1 gene in T cells, reported no deaths within 1 or 2 years. A single trial (NCT01543152) reported serious AEs (SAEs): staphylococcal cellulitis and substance abuse.

The median follow-up time for 21 trials without any results entry in ClinicalTrials.gov and SCD until May 2021 was 17.3 months from the SCD (95% CI 6.2–32.6, range 2.2–100.0 months)

### Characteristics of HGE technologies

The trials mostly tested the intervention in a single health condition (median 1, 95% CI 1.0–1.0, range 1–14), mostly cancers (70.4%) and HIV infection (14.8%) (Table 2).

**Table 2:** Characteristics of genome editing methodologies used in 81 trials registered in WHO ICTRP

Genome editing characteristics	No. (%) of trials
**Platform:**	
ZFN	17 (21.0)
TALEN^a^	1 (1.2)
CRISPR/Cas9	29 (35.8)
TALEN and CRISPR/Cas9^b^	1 (1.2)
Not stated	33 (40.7)
**Testing method:**	
In vivo^c^	9 (11.1)
Ex vivo	72 (88.9)
**Disease applications:**	
HIV infection and AIDS	12 (14.8)
Neoplasms	57 (70.4)
Hematological disorders^d^	8 (9.9)
Metabolic diseases^e^	2 (2.5)
Eye diseases^f^	2 (2.5)
**Edited cells:**	
T cells^g^	24 (29.6)
CAR T cells	37 (45.7)
Tumor infiltrating lymphocytes	2 (2.5)
Stem or progenitor cells^h^	8 (9.9)
Hepatocytes	3 (3.7)
Epithelial cells	3 (3.7)
Human embryos^i^	1 (1.2)
Not stated	3 (3.7)
**Delivery:**	
Adenovirus	1 (1.2)
AAV	4 (4.9)
Lentivirus	2 (2.5)
Lentiviral and electroporation	3 (3.7)
Plasmid	1 (1.2)
mRNA	4 (4.9)
Intratumoral injection	1 (1.2)
Not stated	65 (80.2)

*Abbreviations: AAV* Adeno-associated virus, *CAR* Chimeric antigen receptor, *CRISPR* Clustered regularly interspaced short palindromic repeats, *TALEN* Transcription activator-like effector nuclease, *WHO ICTRP* World Health Organization International Clinical Trial Registry Platform, *ZFN* Zinc finger nuclease

^a^Trial conducted in China, using TALEN in vivo (suppository) in the treatment of HPV-related cervical intraepithelial neoplasia (CIN) (NCT03226470)

^b^Trial conducted in China, using CRISPR/Cas9 and TALEN in vivo (plasmids in gel) in the treatment of HPV-associated CIN (NCT03057912)

^c^HPV-related malignant neoplasm was an investigated condition in 3 trials testing only ZFN, TALEN, or TALEN and CRISPR/Cas9 (NCT02800369, NCT03226470, NCT03057912); hemophilia B or mucopolysaccharidosis in 4 trials using ZFN platform (NCT02695160, EUCTR2017-004805-42-GB, NCT03041324, NCT02702115); and different diseases of the visual system in the remaining 2 trials (and) – one used CRISPR/Cas9 (NCT04560790) and another did not specify the nuclear platform used (NCT03872479)

^d^Including beta-thalassemia, sickle cell disease, and hemophilia B

^e^Referring to mucopolysaccharidosis I and II

^f^ “Blindness, Leber congenital amaurosis 10, vision disorders, hereditary eye diseases, congenital eye disorders, retinal disease/degeneration” noted in NCT03872479; “viral keratitis, blindness, Herpes simplex virus infection” recorded in NCT04560790

^g^Recorded terms as CD4 + T cells, Epstein Barr Virus (EBV)-specific cytotoxic T lymphocytes (CTLs), or only T cells

^h^Including hematopoietic stem/progenitor cells (HSPCs), hematopoietic stem cells (HSCs), and induced hematopoietic stem cells (iHSCs)

^i^CRISPR embryo editing by Chinese scientist He Jiankui, which later resulted in birth (ChiCTR1800019378)

Only 9 (11.1%) trials applied HGE tools directly to a participant’s organism, using nucleases for the treatment of HPV-related malignant neoplasm, hemophilia B, mucopolysaccharidosis, or different eye disorders.

We identified a trial on germline editing (ChiCTR1800019378) involving married Chinese couples with HIV seropositivity and fertility problems. The trial resulted in the birth of twin girls with CRISPR disabled CCR5 gene [28].

CRISPR/Cas9 was the most utilized genome editing platform (35.8%), followed by ZFN (21.0%, Table 2). Out of 33 trials that lacked registration data on the nuclease platform used, 21 (63.6%) were conducted in China.

The description of the delivery platform was absent for the majority of trials (80.2%). Half of trials that provided this information used adeno-associated virus vectors or mRNA (9.9%).

The development of chimeric antigen receptor (CAR) T cells was in the focus of 37 trials (45.7%); 27 of them (73.0%) studied allogeneic CAR T cells, 8 (21.6%) autologous, whilst for 2 trials (5.4%) CAR T cells origin was not precisely stated. B-lymphocyte antigen CD19 was the most commonly targeted protein among CAR therapies (*n* = 18, 48.6%, Table 3). Considering all included trials, knock-out of an immune checkpoint PD-1 was the sole aim for almost a fifth of trials (18.5%, Table 3).

**Table 3:** Registered targets in 81 clinical trials on genome editing in human participants from WHO ICTRP

WHO ICTRP targets	No. (%) of trials
**Single target**	
BCL11A gene	5 (6.2)
CCR5 gene	10 (12.3)
CEP290 gene	1 (1.2)
CISH gene	2 (2.5)
Factor IX gene	2 (2.5)
HBB gene	1 (1.2)
HPV oncogenes E6 or E7	3 (3.7)
IDS gene	1 (1.2)
IDUA gene	1 (1.2)
PDCD1 gene	15 (18.5)
CAR T cells therapy targets:	23 (28.4)
BCMA	3 (3.7)
CD7	2 (2.5)
CD19	12 (14.8)
CD22	1 (1.2)
CD70	2 (2.5)
CD123	2 (2.5)
CS1	1 (1.2)
**Multiple targets**	
PDCD1, NY-ESO-1, TRAC	1 (1.2)
PDCD1, mesothelin (CARTs)	2 (2.5)
PDCD1, CD19 (CARTs)	2 (2.5)
PDCD1, MUC1 (CARTs)	3 (3.7)
CCR5, CD4 (CARTs)	1 (1.2)
CD19 and CD20/CD22 (CARTs)	1 (1.2)
CD19 and HPK1 (CARTs)	1 (1.2)
CD19, CD52, TRAC (CARTs)	1 (1.2)
CD19, B2M, TRAC (CARTs)	1 (1.2)
IL13 zetakine/HyTk (CARTs)	1 (1.2)
CD7 and CD28 (CARTs)	1 (1.2)
**Not stated**	2 (2.5)

*Abbreviations: BCL11A* Mouse B cell lymphoma factor 11A, *BCMA* B cell maturation antigen, *B2M* Beta-2-microglobulin, *CAR* Chimeric antigen receptor, *CARTs* Chimeric antigen receptor T cells, *CCR5* Chemokine receptor 5, *CD* Cluster of differentiation, *CEP290* Centrosomal protein 290, *CISH* Cytokine-induced SH2 protein, *HBB* Hemoglobin subunit beta, *HPK1* Hematopoietic progenitor kinase 1, *HPV* Human papillomavirus, *IDS* Iduronate 2-sulfatase, *IDUA* α-L-iduronidase, *MUC1* Mucin 1, cell surface associated, *NY-ESO-1* New York esophageal squamous cell carcinoma 1, *PDCD1* Programmed cell death 1, *TRAC* T cell receptor alpha chain, *WHO ICTRP* World Health Organization International Clinical Trial Registry Platform

Only 13 (16.0%) out of 81 trials explicitly stated the number of the ethics committee document or date of approval.

### Comparison of registered and published data

Out of 81 trials, 12 trials (14.8%) in ClinicalTrials.gov had results published in a journal (9 full-text and 3 progress reports).

For half of the trials, inclusion criteria were less informative in the publication than in the registry or were not specifically reported (Table 4). A sample size smaller than registered was reported in 5 out of 9 full-text reports. Participant’s age and sex matched in both sources for just over half of the trials.

**Table 4:** Comparison of selected protocol information registered and published for 12 ClinicalTrials.gov trials on genome editing

**Protocol in registry vs. publications**^a^	**No. (%) of trials**
**Sample size**	
Equal absolute number in both sources	4 (33.3)
Smaller sample size in publication	8 (66.7)
Published full-text^b^	5 (41.7)
Published abstract	3 (25.0)
**Eligibility age**	
Congruent in both sources^c^	7 (58.3)
Reported different inclusion age range^d^	5 (41.7)
Published full-text	4 (33.3)
Published abstract	1 (8.3)
**Eligibility sex**	
Congruent in both sources^e^	8 (66.7)
Sex not reported in publication	4 (33.3)
Published full-text	2 (16.7)
Published abstract	2 (16.7)
**Other inclusion criteria**	
Congruent in both sources^f^	3 (25.0)
More informative in registry^e^	3 (25.0)
More informative in article, with changed particular criteria^g^	1 (8.3)
Only diagnosis defined with different levels of details	2 (16.7)
Inclusion criteria not specifically stated in publication^h^	3 (25.0)
**Exclusion criteria**	
Congruent in both sources^f^	1 (8.3)
More informative in registry^i^	4 (33.3)
More informative in publication	1 (8.3)
Exclusion criteria not specifically stated in publication^e^	6 (50.0)
**Primary outcome measures (POMs)**	
Congruent in both sources^j^	9 (75.0)
New outcome introduced in publication^k^	1 (8.3)
POMs not reported clearly and separately from SOMs in article, but all registered POMs congruent to published	2 (16.7)
**Secondary outcome measures (SOMs)**	
Congruent in both sources	2 (16.7)
More informative in registry	1 (8.3)
New outcomes introduced in article	2 (16.7)
Particular outcomes missing in progress report abstract	2 (16.7)
One registered SOM published as POM	1 (8.3)
POMs not reported clearly and separately from SOMs in article, but particular registered SOMs omitted in publication	2 (16.7)
SOMs not registered in ClinicalTrials.gov^e^	2 (16.7)

^a^A total of 12 trials was published: 9 as a full-text and 3 as an abstract of the progress report

^b^NCT03164135: registered vs. published 5 vs. 1; NCT02808442: 13 vs. 7; NCT03655678 and NCT03745287: 45 vs. 1 (reported preliminary results; the first patient included); NCT02746952: 25 vs. 14

^c^Two trials were published in a form of an abstract

^d^NCT03525782: registered 18–70 years, published 36–84; NCT02808442: registered up to 17 years, published 6 months-18 years; NCT03655678 and NCT03745287: registered 12–35, published 18–35; NCT02746952: registered 16–69, published 16–70

^e^One trial was published in a form of an abstract

^f^One abstract was included, with a statement: “Patients were recruited according to the criteria in NCT03525782”

^g^Along with different levels of details, one trial also modified a particular inclusion criterion in the article (NCT02793856): stage IV non-small cell lung cancer and expected life span ≥ 6 months in the registry vs. stage IIIB or IV NSCLC and a life expectancy of over 3 months in the publication

^h^One trial was published as an abstract (NCT02702115), and two other reported a clinical summary of each included patient in the article, including the time of the first diagnosis of target disease, used therapy, intervention protocol, and safety outcomes (NCT03164135 and NCT03399448)

^i^One trial had “prior anti-CD19 cell therapy” as an exclusion criterion in ClinicalTrials.gov, whilst in the published abstract, this treatment was allowed (NCT03939026)

^j^Three trials were published in a form of an abstract

^k^Safety was registered POM, whilst in the article safety along with feasibility were noted (NCT02793856). Feasibility was defined “by sufficient and viable edited T cells being able to be manufactured from the majority of enrolled patients”

The trials registered a median of 1 primary outcome measures (POMs) (95% CI 1–2, range 1–7) and 5.5 secondary (SOMs) (95% CI 2.0–10.6, range 1–21) in ClinicalTrials.gov. AEs were included in the registered POMs for all trials, except one (NCT03164135), which did not include AEs under any outcome measure. The number and description of SOMs matched in published full-text and ClinicalTrials.gov for 2 out of 12 trials (Table 4).

A single trial, declared as a first-in-human phase 1 trial testing CRISPR/Cas9 PD-1-edited T cells in patients with advanced NSCLC (NCT02793856), had results both published and posted in ClinicalTrials.gov. The time frame of AE data collection matched in both sources. The total number of participants with grade 1/2 treatment-related AEs were congruently reported, but SAEs during follow-up were not reported in ClinicalTrials.gov. No death was reported in the registry data element “All-cause mortality”, but the article noted that 11 out of 12 participants died from tumor progression within 2 years.

## Discussion

Our study showed that registered trials using in vivo or ex vivo genome editing technology were mostly prospectively registered and recruiting phase 1 trials, focused mostly on immunotherapy that uses specially altered T cells, CAR T cell therapy, for different types of cancer. Trial results were underreported both in the registries and journal publications. All trials were related to somatic interventions, except a single one that resulted in a birth of two children. This trial involved major ethical violations and a call for international moratorium on the clinical use of human germline editing [29, 30].

In interpreting the findings, the following limitations should be addressed. The HGE Registry is a global registry recently created to track research on human genome editing [26] in the WHO ICTRP, which assembles the trial registration data sets provided by primary registries [31, 32]. However, taking into account that we identified additional 13 trials from two recently published overviews of trials on genome editing, there is a possibility that advanced search tools available at this moment in ICTRP are not sensitive and specific to identify all HGE trials. The number of trials we identified by combining the search of registries and published overviews is the highest among studies published to 2021 [5, 9, 11, 12]. As the trials on HGE were mostly in early phase 1 or phase 1 (51%), which are not subject to recently updated FDAAA registration and results reporting requirements [33], the sample may not be fully representative. We collected trial characteristics by assessing registration protocols that were not always complete.

The finding that only 14% of trials were completed could serve as another explanation for the poor results reporting rate identified in primary registries. It is important to point out that all trials whose status was noted as completed were from ClinicalTrials.gov, the registry with a structured database for results dissemination. Of these completed trials, phase 1 was reported for 64%. However, our findings are consistent with similar studies showing that reporting of results has not yet become routine in research practice [34, 35], especially for trials at earlier stages [36, 37]. Anderson et al. showed that only 13% of 13,327 analyzed trials reported summary results within 1 year after completion [37]. Still, it is difficult to interpret the rate of results reporting for trials involving technologies that are incomparable to conventional drugs, and for which regulatory requirements are still not fully established. In line with that, regulatory reforms for phase 1 trials should be considered in order to optimize the benefits and lessen potential harms from trials including HGE. Among the only 3 completed trials with results registered until May 2021, we showed that none has reported deaths and only one reported SAEs. The AEs underreporting in GT trials was already an issue in 2000 after the first publicly identified death of trial participant [16], when NIH announced that only 39 out of 691 GT-related SAEs in the last 7 years had been reported promptly, as required [38]. Despite 14% trials having completed status, a total of 33 trials (41%) reported SCD before May 2021. Among them, 21 trials (64%) were from ClinicalTrials.gov and remained without submitted results within the median follow-up time of 1.4 years after the SCD. The number of withdrawn, terminated, or trials of unknown status, and identified discrepancies in recruitment status and SCD, all point to challenges in ensuring high quality of data in public registries in general, and for HGE trials in particular. This is in line with the common inadequate updating of registered data [39, 40] even after more than 15 years of the implementation of trial registration [32] and more than 13 years of the legal mandate for results registration [22]. However, the prospective registration rate of almost 70% among HGE trials is higher than the reported 42% prospective registration rate from a 2018 study of 10,500 RCTs published in 2105 journals [41]. Prospective protocol registration is especially important for HGE trials since an expert assessment of publicly available information might prevent unethical, unsafe, illegal, or research without proper scientific justification to be conducted [42].

The discrepancies between the registry and corresponding publications regarding protocol and safety data are another issue of concern. It should be noted that the publication rate of 15% correlates with the percentage of trials with completed status reported under the recruitment information registration field. In a single trial that had both registered and published results, we identified absent reporting of SAEs and all-cause mortality in ClinicalTrials.gov. Moreover, in comparison to registered data, eligibility criteria were mostly underreported in publications. Since incomplete reporting of trial data is often credited to space restrictions in journals [43], the peer-review process may play a more important role in augmenting trial transparency. Nonetheless, it is important to keep in mind that 58% trials had SCD after May 2021, and that a full-text article was identified for only 9 trials at the moment of publication search.

Almost half of the included trials investigated applications of CAR T cells in human subjects. The fast evolution of CAR T cell therapies in both number and type [44] guided the recent inclusion of an annex on CAR T cells in EMA’s guidance on the development of new medicinal products for human use containing genetically modified cells [45]. Universal CAR T cells for specific antigens of interest were generated from allogeneic T cells from healthy donors in more than 70% of the previously mentioned trials. However, these “off-the-shelf” products for large-scale clinical applications are still at their infancy, waiting for the establishment of clinical, industrial, and regulatory standards [46].

Our study showed that CRISPR technology were used more often than ZFNs and TALENs, which could be partially attributed to the difficulty in cloning and protein engineering for ZNF and TALEN, and their less simple and flexible use [12, 47]. The CRISPR technique can be readily and affordably adapted to simultaneously target genome sequence at multiple sites, with remarkable efficiency [48]. Despite that, the potential immunogenicity to CRISPR-Cas9 proteins could be a potential limitation for the use in humans [49], and should be monitored in HGE trials.

It is difficult to discuss the vector systems used for HGE since 80% trials did not register this information. However, among trials with delivery platform recorded, viral vectors were among the most commonly used, probably because they lack the propensity to integrate or reactivate following latency and thus carry a lower risk of delayed AEs [19].

Regarding trial design, the majority of identified ICTRP trials were early-phase trials, initially evaluating safety, tolerability, or feasibility of administration of investigational products [1]. RCTs are generally recommended, according to the EMA’s guidance related to clinical aspects of GT products from 2018, but with acceptable alternatives if appropriately justified [2]. Early-phase design for GT products is more complex than for other product types, as stated in FDA’s guidance released in 2015, and a case-by-case estimation is recommended in the trial planning [1].

Keeping in mind that the use of blinding and the control group in phase 1 or 2 are generally not as crucial as for confirmatory efficacy trial [1], only 4 out of 78 identified interventional HGE trials were blinded. The placebo comparator group was used in 3 trials focused on cancer immunotherapy and included an invasive procedure for the collection of patient’s T lymphocytes. Despite the fact that such a control could help differentiate product-related from procedure-related effects and be relevant for phase 3 trial as well, the use of the invasive procedure in the control group may be an important risk [1].

The finding that more than 80% of trials registered in WHO ICTRP clearly reported non-inclusion of healthy subjects is in line with the recently mentioned FDA’s recommendations [1], regarding unacceptable benefit-risk ratio in most trials with GT products.

The minimal age of participants included in the trials was below 11 years for 15% trials, whilst 56% trials included patients 65 years of age and older. Since specific effects of HGE could be different in children, adults or the elderly, such as the immunogenicity of a viral vector, taking vulnerable populations into consideration during GT development is encouraged in both EMA’s [2] and FDA’s guidance [1].

It is important to point out that pre-market trials of reasonable duration and sample size cannot fully predict the durability of response and the risk for delayed AEs, which makes the clinical review of HGE products more challenging in comparison with conventional drugs [4]. In our study, 3 LTFU studies were identified, but only 1 specified the duration of the extension study, where enrolled subjects will be evaluated for a total of 12 years (NCT04201782). This is in line with recommended “up to fifteen years for genome editing products” in current FDA guidance on LTFU after administration of human GT products [19], and recently updated EMA guidance [45].

Only 20% of analyzed trials were willing to provide an access to IPD and enable the re-use of data. Public posting of informed consent form (ICF) in the trial registry is still not required by the 24-item WHO TRDS, but it might help safeguard subjects from unethical behavior that in the past HGE trials resulted in death or SAEs [50]. In this context, we propose to broaden current WHO registration data elements specifically for HGE trials by including ICF, as well as expanded access information, which should follow the appropriate FDA regulations (21 CFR 312) [51]. Despite the value of data collected during the compassionate use of conventional drugs is mostly considered as limited [52], non-trial preapproval use might provide important information on outcomes and AEs related to this unique class of therapeutics, mostly intended for patient populations that may be small, and whose effects in most cases cannot be reversed [53]. Other steps required to improve the transparency of HGE trials are more demanding, since the WHO ICTRP platform gathers trial registration data sets provided by different primary registries, still not completely complying with the WHO TRDS [54]. However, in 2021 WHO launched recommendations on the governance of HGE on a global scale, including also their trial registration, for which the “traditional” international standards should be particularly adapted [42]. Special emphasis should be put on having the appropriate ethics approval before the inclusion in the WHO HGE Registry. Furthermore, a small expert committee should be established, whose role would be to regularly screen the Registry and assess the compliance of planned and ongoing HGE trials, using a unique registration data set, yet to be standardized [42]. Governments, relevant healthcare and scientific organizations, ethic committees, funders, researchers, journal editors, and reviewers, should put exceptional efforts to enable these potentially life-saving technologies to be responsibly integrated into clinical practice.

## Conclusions

To support safe innovation in this field, product developers should be provided with more specific regulatory guidance reflecting accumulating clinical experience and referring explicitly to HGE products; their development, manufacture, product approval, and follow-up, as well as registration technical requirements. High quality, informative and timely registration of trial protocol and results should be a prerequisite in the clinical regulatory procedure, and the international HGE Registry provided by WHO is a critical and necessary first step toward increasing the transparency of trials on human genome editing, with many fields for advance.

## Supplementary Information

**Additional file 1:** **Supplementary Table 1.** Characteristics of participants included in 81 trials using HGE technologies and registered in WHO ICTRP. **Supplementary Table 2.** Characteristics of conduct of 81 trials using HGE technologies and registered in WHO ICTRP.

## Data Availability

The datasets used and/or analyzed during the current study are available from the corresponding author on reasonable request.

## Abbreviations

AE: Adverse event
CAR: Chimeric antigen receptor
Cas9: CRISPR-associated protein 9
CCR5: Chemokine receptor 5
CFR: The Code of Federal Regulations
CRISPR: Clustered regularly interspaced short palindromic repeats
EMA: European Medicines Agency
FDA: Food and Drug Administration
FDAAA: Food and Drug Administration Amendments Act
GT: Gene therapy
HGE: Human genome editing
ICF: Informed consent form
IPD: Individual participant data
LTFU: Long-term follow-up
NCT number: National Clinical Trial number
NIH: National Institutes of Health
NSCLC: Non-small cell lung cancer
PCD: Primary completion date
PD-1: Programmed cell death protein 1
PDCD1: Programmed cell death protein 1
POM: Primary outcome measure
RCT: Randomized controlled trial
SAE: Serious adverse event
SCD: Study completion date
SOM: Secondary outcome measure
TALEN: Transcription activator-like effector nuclease
UCART19: Allogeneic engineered T-cells expressing anti-CD19 chimeric antigen receptor
WHO ICTRP: World Health Organization International Clinical Trial Registry Platform
WHO TRDS: World Health Organization Trial Registration Data Set
ZFN: Zinc finger nuclease
