# Supplementary material for: Inadequate reporting quality of registered genome editing trials: an observational study
Source: BMC Med Res Methodol. 2022 May 2;22:131. doi: 10.1186/s12874-022-01574-0 (PMC9063127; doi:10.1186/s12874-022-01574-0)
Supplement: Supplementary file 1 — Additional file 1: Supplementary Table 1. Characteristics of participants included in 81 trials using HGE technologies and registered in WHO ICTRP. Supplementary Table 2. Characteristics of conduct of 81 trials using HGE technologies and registered in WHO ICTRP. [file 12874_2022_1574_MOESM1_ESM.docx]

**Additional file 1**

**Supplementary Table 1.** Characteristics of participants included in 81 trials using HGE technologies and registered in WHO ICTRP

**Supplementary Table 2.** Characteristics of conduct of 81 trials using HGE technologies and registered in WHO ICTRP

**Supplementary Table 1.** Characteristics of participants included in 81 trials using HGE technologies and registered in WHO ICTRP

| **Participant characteristics** | **No. (%) of trials** |
| --- | --- |
| **Gender:** |  |
| Male* | 4 (4.9) |
| Female† | 3 (3.7) |
| Both | 74 (91.4) |
| **Healthy volunteers:** |  |
| Stated “yes”‡ | 2 (2.5) |
| Stated “no” | 67 (82.7) |
| No specific field in the registry§ | 12 (14.8) |
| **Minimum age:** |  |
| Provided (median 18, 95% CI: 18-18; range: 6 months-45 years) | 76 (93.8) |
| Not provided | 5 (6.2) |
| **Maximum age:** |  |
| Provided (median 70, 95% CI: 70-70; range: 17-85 years) | 60 (74.1) |
| Not provided | 21 (25.9) |
| **Sample size:\|\|** |  |
| Provided (median 20, 95% CI: 18.0-20.0; range: 0-300) | 81 (100.0) |
| Not provided | 0 (0) |
| **IPD sharing statement:** |  |
| Stated “yes” | 16 (19.8) |
| Stated “no” | 22 (27.2) |
| Stated “undecided” | 14 (17.3) |
| Plan not provided | 29 (35.8) |

*Abbreviations:* HGE, human genome editing; IPD, individual participant (patient) data; WHO ICTRP, World Health Organization International Clinical Trial Registry Platform.

*Two trials were focused on prostate cancer (NCT03525652, NCT02867345), and the other two on hemophilia B (NCT02695160, EUCTR2017-004805-42-GB).

†In all three trials (NCT02800369, NCT03226470, and NCT03057912) human papillomavirus-related malignant neoplasm was the only registered condition.

‡NCT04026100 and NCT03752541 stated to be including healthy volunteers under the appropriate field in CT.gov, but their inclusion criteria comprised the confirmed diagnosis of the target disease.

§Trials registered within the Chinese Clinical Trial Registry.

||Used actual enrollment when available, in all other cases used estimated enrollment.

**Supplementary Table 2.** Characteristics of conduct of 81 trials using HGE technologies and registered in WHO ICTRP

| **Trial conduct characteristics** | **No. (%) of trials** |
| --- | --- |
| **Recruitment status:** |  |
| Not yet recruiting | 9 (11.1) |
| Recruiting | 42 (51.9) |
| Enrolling by invitation | 3 (3.7) |
| Active, not recruiting | 4 (4.9) |
| Terminated* | 1 (1.2) |
| Completed | 11 (13.6) |
| Withdrawn* | 5 (6.2) |
| Unknown | 6 (7.4) |
| **Recruiting locations:** |  |
| United States (US) | 25 (30.9) |
| US and other state(s)† | 12 (14.8) |
| United Kingdom (UK) | 1 (1.2) |
| China | 39 (48.1) |
| Not provided | 4 (4.9) |
| **Registration date:**‡ |  |
| Provided (median 2018, 95% CI: 2017-2018 ; range: 2009-2020) | 81 (100.0) |
| Not provided | 0 (0) |
| **Study start date:**§ |  |
| Provided (median 2018, 95% CI: 2017-2019 ; range: 2009-2021) | 81 (100.0) |
| Not provided | 0 (0) |
| **Recruitment time:**\|\| |  |
| Before registration | 26 (32.1) |
| After registration | 55 (67.9) |
| **Primary completion date:**¶ |  |
| Provided (median 2021, 95% CI: 2020-2021; range: 2013-2040) | 68 (84.0) |
| No specific field in the registry | 13 (16.0) |
| **Study completion date:**** |  |
| Provided (median 2021, 95% CI: 2021-2022; range: 2013-2040) | 80 (98.8) |
| Not provided | 1 (1.2) |
| **Study results:** |  |
| Recorded†† | 3 (3.7) |
| Not recorded | 78 (96.3) |
| **Sponsor:** |  |
| Pharmaceutical industry | 36 (44.4) |
| Community-based organization | 24 (29.6) |
| University | 21 (25.9) |
| **Investigator(s):** |  |
| Provided | 56 (69.1) |
| Not provided | 25 (30.9) |
| **Collaborator(s):** |  |
| Provided | 32 (39.5) |
| Not provided | 36 (44.4) |
| No specific field in the registry‡‡ | 13 (16.0) |
| **Responsible party:** |  |
| Provided | 68 (84.0) |
| No specific field in the registry‡‡ | 13 (16.0) |

*Abbreviations:* HGE, human genome editing; WHO ICTRP, World Health Organization International Clinical Trial Registry Platform.

*NCT03399448 was terminated “to pursue other targets by sponsor”. Among 5 withdrawn trials, 3 listed “no funding” as an explanation (NCT02863913, NCT02867332, NCT02867345), 1 trial listed “sponsor's decision and not a consequence of any safety concern” (NCT04106076), and the remaining one, led by Chinese scientist He Jiankui (ChiCTR1800019378), had noted that “the original applicants cannot provide the individual participants data for reviewing.”

†Among 12 trials, 1 trial (8.3%) registered US with UK, 1 (8.3%) US along with EU states, 2 (16.7%) US and non-EU states, and remaining 8 (66.7%) US with EU state(s), along with UK or non-EU state(s).

‡Used “First Submitted Date” in CT.gov, “Date on which this record was first entered in the EudraCT database” in EudraCT, and “Date of Registration” in ChiCTR.

§Used “Study Start Date” in CT.gov, “Start Date” in EudraCT, and the first date within the field “Recruiting time” in ChiCTR.

||Evaluated using study start dates and registration dates mentioned previously.

¶The date when all required data collection for all the primary outcome measures is completed; used for calculation of median results reporting time. The field “Primary Completion Date” was required only in CT.gov; hence, trials from ChiCTR (n=12) and EudraCT (n=1) did not have this information provided.

**The date based on the collection of final data for a clinical study and the last participant’s visit to a trial center. Used “Study Completion Date” in CT.gov, and second date within the field “Study execute time” in ChiCTR. Trial from EudraCT was lacking this information (n=1).

††All 3 trials reported no deaths within the time frame of 1 and 2 years in 26 (NCT01543152), 8 (NCT02225665) and 12 participants (NCT02793856).

‡‡Entries specifically required only by CT.gov.
